# Efficient chromosomal gene modification with CRISPR/*cas9* and PCR-based homologous recombination donors in cultured *Drosophila* cells

**DOI:** 10.1093/nar/gku289

**Published:** 2014-04-19

**Authors:** Romy Böttcher, Manuel Hollmann, Karin Merk, Volker Nitschko, Christina Obermaier, Julia Philippou-Massier, Isabella Wieland, Ulrike Gaul, Klaus Förstemann

**Affiliations:** 1 Gene Center and Dept. of Biochemistry, Ludwig-Maximilians-Universität, Feodor-Lynen-Str. 25, D-81377 München, Germany; 2 Center for Integrated Protein Science Munich (CIPSM), Ludwig-Maximilians-Universität, Feodor-Lynen-Str. 25, D-81377 München, Germany

## Abstract

The ability to edit the genome is essential for many state-of-the-art experimental paradigms. Since DNA breaks stimulate repair, they can be exploited to target site-specific integration. The clustered, regularly interspaced, short palindromic repeats (CRISPR)/*cas9* system from *Streptococcus pyogenes* has been harnessed into an efficient and programmable nuclease for eukaryotic cells. We thus combined DNA cleavage by *cas9*, the generation of homologous recombination donors by polymerase chain reaction (PCR) and transient depletion of the non-homologous end joining factor *lig4*. Using cultured *Drosophila melanogaster* S2-cells and the phosphoglycerate kinase gene as a model, we reached targeted integration frequencies of up to 50% in drug-selected cell populations. Homology arms as short as 29 nt appended to the PCR primer resulted in detectable integration, slightly longer extensions are beneficial. We confirmed established rules for *S. pyogenes cas9* sgRNA design and demonstrate that the complementarity region allows length variation and 5′-extensions. This enables generation of U6-promoter fusion templates by overlap-extension PCR with a standardized protocol. We present a series of PCR template vectors for C-terminal protein tagging and clonal *Drosophila* S2 cell lines with stable expression of a myc-tagged *cas9* protein. The system can be used for epitope tagging or reporter gene knock-ins in an experimental setup that can in principle be fully automated.

## INTRODUCTION

It has taken only a surprisingly short time between the initial discovery of CRISPR/*cas* systems as a phage defense mechanism (1) based on RNA-programmed (2), sequence-specific nucleases (3) and the development of a specific subtype into a versatile tool for genome editing (4–6). It has since been successfully applied to budding yeast (7), *Caenorhabditis elegans* and other nematodes (8–15), *Drosophila* (16–21), *Arabidopsis, Nicotiana, Sorghum* and *Oryza* (22–26), zebrafish (27–33), *Xenopus* (34), mice (32,35–41), rats (42), rabbits (43), beef (44), cynomolgus monkey (45) and human cells (46–50)—all within little more than a year. There are few examples of comparable success of a new technology. The broad applicability—from yeast to plants and mammals—indicates that the system is indeed highly efficient despite the fact that it has not evolved to cleave DNA associated with eukaryotic nucleosomes. Researchers are now working to improve cleavage specificity of the system (51–55) and to make use of the programmable, sequence-specific binding for other purposes than DNA cleavage (56–62).

Several of the above-cited studies already made use of CRISPR/*cas9* induced DNA breaks to stimulate repair via an experimentally provided homologous recombination (HR) donor construct. These editing tools were either large plasmid-based constructs with long regions of sequence homology (kb range) or single-stranded oligonucleotides with a short region of sequence homology. The plasmid constructs usually template repair efficiently and can transfer large tag sequences, but their generation is time-consuming. Single-stranded oligonucleotides are conveniently produced via chemical synthesis but due to size restraints their ‘coding capacity’ is limited; they are most useful for specifying point mutations in active sites or introduction of e.g. *loxP* or *attP* sites (reviewed in (63)). An approach that unites the convenience of oligonucleotide ordering with the capacity to introduce large tags has been developed in budding yeast (64): a series of plasmid templates combines e.g. epitope tags and a selectable marker, which are then amplified with flanking primers that specify the desired integration site via appended homology arms. The presumably lower integration efficiency of the short homology arms is outweighed by the very efficient selection that is possible with microorganisms.

Inspired by the success of the CRISPR/*cas9* technology and the ease of protein tagging available in budding yeast, we attempted to use polymerase chain reaction (PCR)-based homology donors for genome editing in cultured *Drosophila* S2 cells. Efficient selection of stably transformed cells via a blasticidin resistance gene and stable expression of the *cas9* nuclease allowed us to readily recover cells with targeted integration of a donor construct at the phosphoglycerate kinase (PGK) locus flanked by as little as 29 nt of sequence homology on either end. Repair via the non-homologous end joining (NHEJ) pathway is a competing reaction for this purpose, eventually leading to mutation of the CRISPR target site without integration of the donor construct. Consistent with this notion, we find that depletion of the *lig4* gene (65) via RNA interference prior to induction of the DNA break substantially increased the proportion of cells harbouring the desired integrates, reaching a level of up to 50% of the blasticidin resistant cell population. Similar effects have been observed in *lig4* mutant *Drosophila* embryos with Zn-finger induced DNA double-strand breaks (DSBs) (66). For many purposes this level may already be sufficient; furthermore, we found it straightforward to derive cell clones with uniform expression by serial dilution. Compared with transient transfections or stable cell lines generated by conventional approaches, our technique is not only more convenient but also avoids potential artifacts due to heterogeneous and exaggerated expression levels of the tagged protein. The approach we are presenting here is based on transfection of PCR products and *in vitro* transcribed guide RNA. The latter can be replaced by U6-promoter-sRNA template fusions also generated by PCR according to a standardized protocol. However, in this case spontaneous integration of the gRNA expression cassette may lead to permanent activation of the *cas9* nuclease and, consequently, a high potential for off-target cleavage events.

## MATERIALS AND METHODS

### Molecular biology techniques

The cleavable double-GFP reporter was generated by inserting a *Not*I/*Xba*I digested PCR product of green fluorescent protein (GFP) obtained with primers 5′-atGCGGCCGCGTGAGCAAGGGCGAGGAGCT-3′ and 5′-TAtctagaggccgctTTACTTGTACAGCTCGTCCATGC-3′ into *Not*I/*Xba*I cut pKF63 (67). The resulting plasmid was opened at the *Not*I-site in between the two GFP moieties and the annealed oligonucleotides 5′-ggccTAGGGATAACAGGGTAATgc-3′ and 5′- ggccgcATTACCCTGTTATCCCTA-3′ were inserted; the orientation was determined by sequencing. The final plasmid, pKF251, has a ubiquitin promoter, a fully functional copy of myc-GFP followed by the *I-Sce*I recognition site and a second GFP that lacks an N-terminal methionine. We amplified the *I-Sce*I ORF from a fly strain harbouring a heat-shock promoter driven *I-Sce*I transgene (68) with primers 5′-caGGTACCTAATCCAAAATGTTCATGCCTTCTTCTTTTTCC-3′ and 5′-atGCGGCCGCTTATTTCAGGAAAGTTTCGGAGGAG-3′, digested the PCR product with *Kpn*I/*Not*I and inserted the fragment into *Kpn*I/*Not*I-cut pCASPER tub (69) yielding pKF257. This vector leads to expression of the *I-Sce*I nuclease under the control of the α-tub84 promoter.

The *cas9* gene with humanized codon bias (47) was amplified with primers 5′-ATggtaccTAATCCAAAatggaacagaaactgattagcgaagaagacctgGACAAGAAGTACTCCATTGG-3′ (appending a myc-tag at the N-terminus) and 5′-atgcggccgcTCACACCTTCCTCTTCTTCTTG-3′, digested with *Kpn*I/*Not*I and ligated into correspondingly cleaved pKF257 (see above). Furthermore, an *attB* sequence was inserted into a unique *Nde*I site in this plasmid with annealed oligos 5′-TATGgggtgccagggcgtgcccttgggctccccgggcgcgta-3′ and 5′-TAtacgcgcccggggagcccaagggcacgccctggcacccCA-3′ resulting in plasmid pRB14 (expression of myc-*cas9*).

To generate a vector that allows for expression of the site-specific recombinase FLP from *Saccharomyces cerevisiae*, the gene was PCR amplified from yeast genomic DNA (strain W303) using primers 5′-atggatccATGCCACAATTTGGTATATTATGTAAAAC-3′ and 5′-atgcggccgcTTATATGCGTCTATTTATGTAGGATGAAAG-3′, then inserted into *Bam*HI/*Not*I cut pKF63 yielding plasmid pMH5. The insert was sequence verified.

To generate sgRNA templates for *in vitro* transcription via PCR, the oligonucleotide 5′-GTTTTAGAGCTAGAAATAGCAAGTTAAAATAAGGCTAGTCCGTTATCAACTTGAAAAAGTGGCACCGAGTCGGTGC-3′ served as PCR template with a sense primer containing the T7 promoter and targeting sequence (e.g. 5′-taatacgactcactataGCCTAGGGATAACAGGGTAATGgttttagagct-3′) and 5′-GCACCGACTCGGTGCCACT-3′ as antisense primer. For the 3′-extended CRISPR's, oligo 5′-GTGAGCAAGGGCGAGGAGgcaccgactcggtgccact-3′ served as antisense PCR primer. Details on CRISPR target site oligonucleotides employed are given in Figure 2A and the supplementary information. *In vitro* transcription was performed as previously described for dsRNA (70), the sgRNA products were purified via a Qiagen PCR purification kit. PCR amplification of the U6-C promoter with a T7-extension was achieved with oligonucleotides 5′-GCTCACCTGTGATTGCTCCTAC-3′ and 5′- atagtgagtcgtattaAACGACGTTAAATTGAAAATAGGTCTA-3′. The PCR product was cloned into pJet1.2 resulting in plasmid pRB17 and sequence verified. Overlap-extension PCR was performed with 1 μl of sgRNA *in vitro* transcription template (see above) and 1 μl of a 10 ng/μl dilution of pRB17 per 50 μl PCR. The primers for this PCR were 5′-GCTCACCTGTGATTGCTCCTAC-3′ and 5′-gcttattctcAAAAAAGCACCGACTCGGTGCCACT-3′ (to introduce a pol-III termination signal at the end).

**Figure 1.**
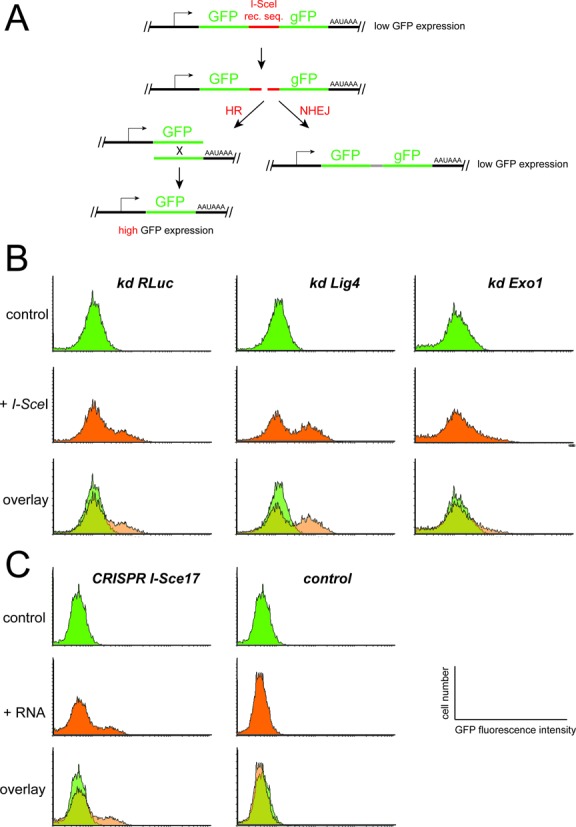
*I-Sce* I and CRISPR-mediated cleavage of chromosomal DNA occur with comparable efficiency. (**A**) Schematic representation of the double-GFP reporter construct. Note that the first copy of GFP is complete and therefore functional, and the second copy only lacks the initiating methionine (symbolized by the lower case g). The non-rearranged reporter leads to low GFP fluorescence in clonally selected, stable cell lines. Upon cleavage at the intervening *I-Sce*I recognition sequence, repair may occur either via homologous recombination (HR, left side) or non-homologous end joining (NHEJ, right side). While HR rearranges the locus and results in high GFP expression, NHEJ leads to short deletions but retains the low level of GFP fluorescence. (**B**) Depletion of *lig4* (an essential NHEJ factor) and *exo1* (an essential HR factor) followed by transfection of an *I-Sce*I nuclease expression vector demonstrates the preferred outcome of reporter fluorescence when either pathway is suppressed. Knock-down of *Renilla* luciferase served as a control. (**C**) In clonally selected stable cell lines that express a myc-tagged version of the *cas9* nuclease protein in addition to the reporter construct, transfection of an *in vitro* transcribed, cognate CRISPR RNA (see Figure 2A for sequence details, I-Sce17 refers to the 17-nt-long CRISPR targeting region) leads to a comparable extent of DNA cleavage as the *I-Sce*I nuclease itself (as judged by the proportion of GFP^high^ cells). A control RNA (CRISPR target sequence 5′- GCGGTGGACCAGCTGCAGC-3′) that does not target *cas9* to our reporter did not lead to detectable cleavage and rearrangement.

**Figure 2.**
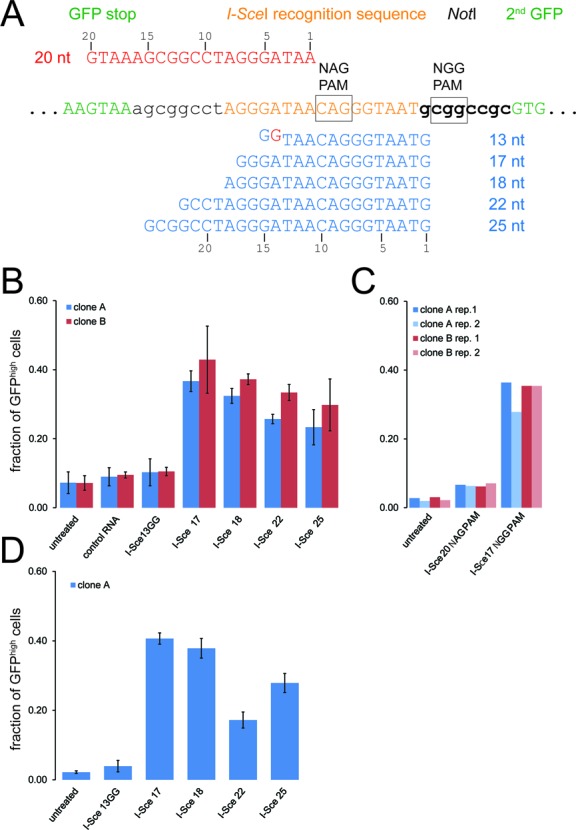
Quantitative assessment of *cas9*-mediated cleavage in living cells using various CRISPR RNA guides. (**A**) Sequence detail of the *I-Sce*I recognition site region in our reporter construct. The length variant CRISPR RNAs with an NGG trinucleotide protospacer-associated motif (PAM) are shown in blue below the reporter sequence and the CRISPR RNA for testing cleavage efficiency at the 5'-NAG-3' trinucleotide PAM is shown in red above the reporter sequence. (**B**) Quantitative analysis of CRISPR cleavage activity according to targeting sequence length using our HR reporter system. The proportion of GFP^high^ cells was determined via two-dimensional analysis of fluorescence-activated cell sorting data (side scatter vs. GFP fluorescence) as this enables more reliable separation of the two populations. Two independent cell clones expressing myc-*cas9* and the double-GFP reporter were analyzed; the data are presented as the mean ±SD of three independent biological replicates. (**C**) Quantitative analysis of CRISPR cleavage activity with an NAG trinucleotide PAM. The experiment was performed essentially as described in B, the I-Sce17 NGG CRISPR serves as comparison. Cleavage activity towards an NAG PAM is detectable but occurs with clearly lower efficiency. Two cell clones were tested in two independent biological replicates each. (**D**) Extending the CRISPR RNA at its 3′-end does not impair cleavage activity. The experiment was performed and analyzed as in B but a CRISPR RNA with a 3′-extension harboring 18 nt of sequence homology downstream of the CRISPR target site was employed. This extension did not impair cleavage activity (compare with B), but also did not rescue the apparent defect of the I-Sce13GG RNA construct either.

A *copia-Bsd-eGFP* fusion gene was constructed by amplifying the copia promoter from pDrBB2 (Addgene) with primers 5′-GCgaattcCTGTTGGAATATACTATTCAAC-3′ and 5′-GCggtaccTCCTTTCTTAATAAATAAATAAATAG-3′, the *bsd* gene from pCMV/Bsd (Invitrogen) with primers 5′-GCaccggtATGGCCAAGCCTTTGTCTCA-3 and 5′-GCccatggtGCCCTCCCACACATAACCA-3′ and the eGFP reporter from pEGFP-N1 (Addgene) with primers 5′-GCccATGGTGAGCAAGGGCGAG-3′ and 5′-GCgctagcTTACTTGTACAGCTCGTCCA-3′, then inserting them via restriction digest into a pUC19 derivative.

Template plasmids for HR-mediated protein tagging were generated by amplifying the *copia-Bsd* portion of the fusion gene described above with primers 5′-GATTATAAAGATGATGATGATAAAactagtGAAGTTCCTATACTTTCTAGAGAATAGGAACTTCAAATAAAcatatgCTGTTGGAATATACTATTCAAC-3′ and 5′-atCATATGTTAGAAACAAATTTATTTTTAAAGTTTTATTTTTAATAATTTctaGCCCTCCCACACATAACCAGA-3′ to remove the GFP ORF, add a 5′-FRT sequence, a stop codon and a short 3′-UTR. The product was re-amplified with the same sense primer and 5′-GAAGTTCCTATTCTCTAGAAAGTATAGGAACTTCcatatgttagaaacaaatttatttttaaag-3′ to append a downstream FRT site, then inserted via blunt-end cloning into pJet2.1. The orientation was determined by sequencing. This plasmid served as the basis for tag insertion between an *Xho*I and *Spe*I site. We used primers 5′-tactcgagATGGTGAGCAAGGGCGAGGAG-3′ and 5′-taactagtTTACTTGTACAGCTCGTCCATG-3′ to amplify the coding sequences of EGFP resulting in plasmid pMH3. A double FLAG-tag template was constructed by ligating the annealed oligonucleotides 5′-tcgagGATTATAAAGATGATGATGATAAAtccggagccGATTATAAAGATGATGATGATAAATGAa-3′ and 5′-ctagtTCATTTATCATCATCATCTTTATAATCggctccggaTTTATCATCATCATCTTTATAATCc-3′ into the base vector resulting in plasmid pMH4. The TwinStrep-tag sequence was inserted via the annealed oligonucleotides 5′-tcgagAGCGCTTGGAGCCACCCGCAGTTCGAGAAAGGTGGAGGTTCCGGAGGTGGATCGGGAGGTGGATCGTGGAGCCACCCGCAGTTCGAAAAATGAa-3′ and 5′-ctagtTCATTTTTCGAACTGCGGGTGGCTCCACGATCCACCTCCCGATCCACCTCCGGAACCTCCACCTTTCTCGAACTGCGGGTGGCTCCAAGCGCTc-3′ resulting in plasmid pIW1.

**Figure 3.**
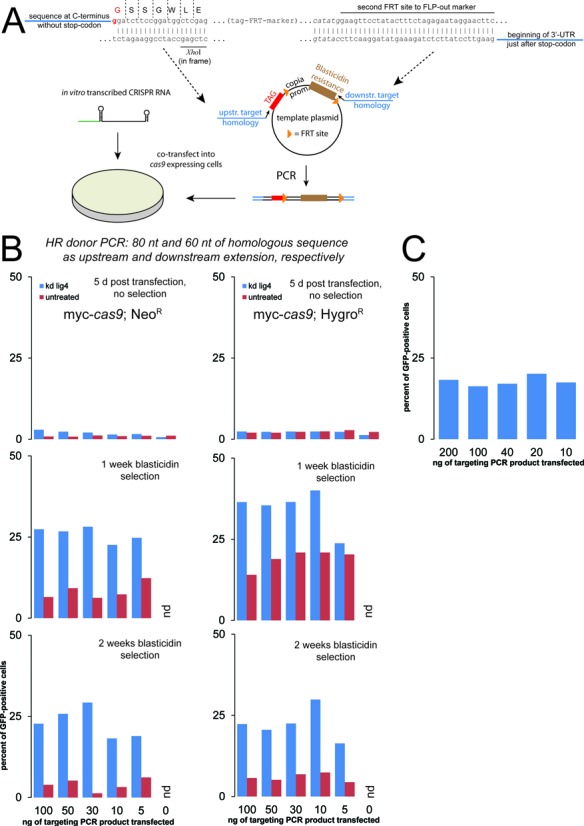
Using CRISPR-mediated induction of DNA double-strand breaks for genome editing via HR. (**A**) Schematic representation of the targeting construct design and transfection; the length of the homology arms appended via the PCR primers was 80 nt upstream and 60 nt downstream. Further details, including targeting design, are provided in the supplementary information. (**B**) Assessment of targeting efficiency using the integration of a GFP moiety at the C-terminus of PGK. The three panels represent flow cytometry analysis 5 days after the transfection (top) and after one and two weeks of blasticidin selection (middle and bottom, respectively). The quantity of PCR product transfected (indicated below the bottom panel) refers to the amount applied to one well in a 96-well plate, the amount of sgRNA employed was 100 ng per well. We compared two stable cell clones expressing myc-*cas9* that were independently selected (neomycin and hygromycin resistance) either as untreated cells (red bars) or as cells where the NHEJ factor lig4 had been depleted before transfection (two sequential dsRNA treatments, blue bars). The slightly higher proportion of cells detected as GFP positive after the first split in blasticidin-containing medium may have been caused by a not yet complete antibiotic selection process; non-integrating but transfected cells will be transiently resistant to blasticidin. Since the GFP fluorescence is low (endogenous levels), it is challenging to completely discriminate dying cells from weak GFP positive cells. Note that in the control transfection without any targeting PCR product, no cells could be measured upon selection (nd = none detected). (**C**) An independent experiment with the neomycin selected myc-*cas9* cell clone yielded comparable results. For this experiment, only one dsRNA treatment to deplete *lig4* was performed; this may explain the slightly lower efficiency compared with B.

PCR amplification of targeting constructs (HR donors) was performed with one of the plasmids described above as template and the primers annealing with their 3′-end sequences of …ggatcttccggatggctcgag-3′ and …gaagttcctattctctagaaagtataggaacttccatatg-3′. Further details are provided in Figure 3 and the supplementary information. For PGK targeting, the primers were 5′-CGTCTCCACCGGAGGCGGCGCTTCGCTGGAGCTCCTGGAGGGCAAGACACTGCCAGGCGTGGCTGCATTGACCAGCGCCggatcttccggatggctcgag-3′ and 5′-TGGTTTGTGCTTACAAGGTAAACGATGCGATTAACATTAATATACCGTATATATGTACGCgaagttcctattctctagaaagtataggaacttccatatg-3′ (80 and 60 nt homology) or 5′-TGCCAGGCGTGGCTGCATTGACCAGCGCCGgatcttccggatggctcgag-3′ and 5′-TAACATTAATATACCGTATATATGTACGCgaagttcctattctctagaaagtataggaacttccatatg-3′ (29 nt homology). PCR products were purified via Qiagen PCR purification columns before transfection. The sgRNA template primer for PGK was 5′-taatacgactcactataGTATATATGTACGCTTAGGCGCgttttagagctag-3′. For tagging of Tubulin 56D, the HR donor primers were 5′-ATGAACGATCTGGTGTCCGAGTACCAGCAGTACCAGGAGGCCACCGCCGACGAGGACGCCGAGTTCGAGGAGGAGCAGGAGGCTGAGGTCGACGAGAACggatcttccggatggctcgag-3′ and 5′-TACAACTGAATTCATTTGTTCTCGTTTCATTTTTTTTCGCAAAACATTGATCGAGAATTCGATTGATTTCCGATTCGAATGAAGTTCCTATTCTCTAgaaagtataggaacttccatatg-3′; the sgRNA template primer was 5′-taatacgactcactataGACGAGAACTAAATTCGAATgttttagagctag-3′. For tagging of the *blanks* gene, the HR donor primers were 5′-ATCCAAGAAGGCAGCCCGTTACAAACTATCCGCTTTAGTTTGTAACAAACTATTTGGAACCGACTATCCACAAAAAGgatcttccggatggctcgag-3′ and either 5′-GAATGCCGCTAATACGTTTTAAAGTACTACCGGGTGCCCAGAATCATGTGATGTTTTCGTGAAGTTCCTATTCTCTAgaaagtataggaacttccatatg-3′ (wild-type sequence) or 5′-GAATGCCGCTAATACGTTTTAAAGTACTACCGGGTGCCCAGAGTTATGTGATGTTTTCGTGAAGTTCCTATTCTCTAgaaagtataggaacttccatatg-3′ (two point mutations in 3′-UTR located CRISPR target site underlined); the sgRNA template primer was 5′-taatacgactcactatagACGAAAACATCACATGATTCTgttttagagct-3′.

Primers to verify integration at the PGK locus were 5′-GTGGCCGCCACCAAGAACG-3′ (PGK-specific, sense) and 5′-GTAGGTTGAATAGTATATTCCAACAGCATATG-3′ (within the copia-promoter, antisense). To detect FLP-mediated excision of the resistance cassette, we used primers 5′-ggatcttccggatggctcgag-3′ (common linker between target protein and tags, sense) and 5′-CCTCAATGGACAGCAACTTTGCCC-3′ (PGK specific, downstream antisense).

All oligonucleotides for cloning and generation of targeting PCR products were ordered from Eurofins/MWG (Ebersberg, Germany) at the appropriate synthesis scale and only standard purification (high purity salt free or HPSF).

The relevant plasmids will be made available through Addgene.

The dsRNA for knock-down was prepared as previously described (71) with PCR primers containing a T7 promoter. For *lig4* RNAi the primers were 5′-taatacgactcactatagggCCCAATGATCCAAAGTGTTTTTGCA-3′ and 5′-taatacgactcactatagGGAAGTAGGATGCCTTCGCGA-3′, for *exo1* RNAi the primers were 5′-taatacgactcactatagggCCGAAGTCGCTTCTTCGCCAC-3′ and 5′-taatacgactcactatagggGGACAGGTTTCGTCTGGAAGC-3′. The *Renilla* luciferase control construct has been described before (70).

### Cell culture and transfections

Cells were cultured in standard Schneider's medium supplemented with 10% fetal bovine serum (both Bio&Sell, Nürnberg/Germany). Transfections were performed with Fugene HD (Promega) and selection of hygromycin or neomycin resistant cells was done as previously described (72). Selection of blasticidin-resistant cells was achieved at a concentration of 25 μg/ml blasticidin-S (Life Technologies). During selection, cells were split between 1:5 and 1:10 once per week. We employed a Becton Dickinson FACSCalibur flow cytometer equipped with a 96-well plate autosampler for quantification of PGK-GFP expression. Data analysis was performed using flowing software version 2.5.0 (http://www.flowingsoftware.com/). RNAi was induced by adding dsRNA to the growth medium at a concentration of 10 μg/ml. In our experience, serum withdrawal is not required for efficient RNAi in S2-cells. Four days after induction of RNAi the cells were either transfected or split 1:10 for a second round of RNAi targeting the same gene. Fluorescence microscopy was performed on a Leica TCS SP2 confocal microscope.

## RESULTS

### The *Streptococcus pyogenes cas9* nuclease efficiently cleaves chromosomal DNA of cultured *Drosophila* cells

Our goal was to introduce a protein tag at the C-terminus with only short regions of homology, therefore selection of suitable CRISPR target sites was very much restrained. We thus tested whether variations of CRISPR RNA length can be tolerated. To this end, we constructed a direct repeat of two GFP coding sequences interrupted by one copy of the *I-Sce*I nuclease recognition sequence (Figure 1A). The reporter is conceptually equivalent to comparable systems developed for mammalian cell culture (73). Although in our case the first GFP sequence is complete (while the second one lacks the original ATG start codon), the complete reporter cassette only yields moderate green fluorescence in S2-cells. Upon cleavage by the *I-Sce*I nuclease, repair via HR between the two GFP sequences can occur (most likely via the single-strand annealing pathway). This leads to a substantial increase in GFP fluorescence, which can be quantified by flow cytometry in stable cell clones. Apparently, in the complete reporter cassette the long 3′-UTR, comprising the second GFP copy, results in inefficient protein expression. PCR analysis of genomic DNA confirmed rearrangement of the locus (data not shown). Thus, the conversion to high GFP expressing cells in our reporter culture indicates DNA cleavage. As an alternative to HR, the NHEJ pathway may also repair the cleaved reporter. In this case, however, no increase in GFP expression will be observed. By depleting the cellular pool of either DNA ligase 4 (*lig4*), an essential enzyme for NHEJ, or the HR factor exonuclease 1 (*exo1*) (74) via RNA interference, the corresponding pathways can be substantially repressed. Subsequent transfection of the *I-Sce*I nuclease and analysis of GFP expression by flow cytometry correctly reflected action of the corresponding repair pathway (Figure 1B). According to preliminary PCR analysis (not shown), it is indeed the repair via HR/single-strand annealing that leads to cells expressing high levels of GFP, but a more thorough analysis of the repair mechanism(s) is required and will be presented elsewhere. For the purpose of this manuscript, the important feature of the reporter is that a rearrangement of the locus is quantifiable and triggered only upon DNA cleavage. Thus, our GFP–*I-Sce*I seq.–GFP reporter construct can be used to indirectly estimate and compare cleavage efficiencies.

We introduced an expression construct for *cas9* with humanized codon bias (*hcas9*, (47) with an additional myc-tag appended) and selected cell clones expressing both, myc-*hcas9* and the GFP–*I-Sce*I seq.–GFP reporter. In these cells, introduction of an *in vitro* transcribed CRISPR RNA with corresponding sequence can direct *cas9*-mediated cleavage of the GFP reporter (Figure 1C). The template for *in vitro* transcription of the CRISPR RNA can be conveniently generated by PCR (see supplementary information for details).

### Length variations of the CRISPR sgRNA are tolerated

We used this system to compare cleavage efficiencies between CRISPR RNA sequences with the capacity to form RNA–DNA hybrids between 13 nt and 25 nt in length. The minimal target length construct contained two additional guanosines at the 5′-end to ensure efficient transcription by T7 RNA polymerase. Among the RNA–DNA hybrid lengths tested, we found that sequences between 17 nt and 25 nt efficiently programmed *cas9* to cleave the reporter DNA with no negative effects induced by shortening of the targeting sequence length to less than 21 nt; in fact, a trend towards higher cleavage efficiencies with 17 nt and 18 nt targeting length is apparent but should be validated with an independent sequence. The shortest construct, however, did not result in detectable cleavage and repair (Figure 2B). Recent reports have described that an NAG trinucleotide may also serve as PAM for *S. pyogenes cas9*, though cleavage efficiency is only about one-fifth of the one observed with a GG dinucleotide (75–77). When cleavage of our GFP–*I-Sce*I seq.–GFP reporter was programmed adjacent to an NAG PAM (see Figure 2A) we observed indeed that recombination-mediated repair is induced, albeit with much lower efficiency (Figure 2C). The 3′-end of the artificial sgRNA scaffold we employ to program *cas9* cleavage specificity has been shortened relative to the naturally occurring CRISPR/tracr-RNA pair (4,47). We thus reasoned that it may be extended in order to append further functional sequences, e.g. a longer stretch of double-stranded RNA or chemical modifications to enhance cellular uptake, if desired. We first prolonged the 3′-end with a short sequence homologous to the reporter sequence downstream of the *I-Sce*I cleavage site (we chose the opposite DNA strand). The rationale was to test whether this addition could restore efficient cleavage of our shortest CRISPR targeting RNA construct by providing the potential to form additional base-pairs 3′ to the PAM. While cleavage of our reporter occurred with the same efficiency for the 3′-extended, 17–25 nt targeting region CRISPR RNAs, the shortest construct still did not show any detectable cleavage activity (Figure 2D).

Cleavage by the *I-Sce*I meganuclease is of comparable efficiency and both systems are most likely limited by the transfection efficiency (we routinely obtain 60–70%). The RNA–DNA hybrid length can be considerably more variable than what is observed in the original organism *S. pyogenes*; changing its length between 17 nt and 25 nt did not reduce cleavage efficiency. Since both the T7 RNA polymerase promoter we exploited for *in vitro* transcription and the frequently employed U6 snRNA promoter for *in vivo* transcription of the CRISPR RNA initiate most efficiently with a guanosine nucleotide, our observation expands the repertoire of biochemically accessible CRISPR target sites within the genome. This is consistent with mutational studies demonstrating that cleavage specificity is primarily determined by the first 12 nt of the CRIPSR targeting portion (counting from the NGG PAM) (4,48,51,75–78). Our observation agrees with previous reports demonstrating that the CRISPR RNA can be extended at its 3′-end (61,78). A contribution of further guideRNA-target DNA base pairs that could rescue the activity of an excessively shortened guide RNA was however not observed with the particular extension we chose. While our manuscript was in revision, Fu et al. (55) reported the use of truncated guide RNAs in human cells. Consistent with our results, guide RNA lengths of 17 and 19 nt showed full cleavage activity while a 15 nt guide RNA appeared to be inactive in their experiments. Furthermore, variations of CRISPR guide RNA length have been tolerated to a certain extent when the CRISPR/*cas9* system was repurposed into a tool for gene regulation (61). Extension of the sgRNA was also tested when Ren et al. used microinjection of DNA templates for sgRNA transcription in *Drosophila* embryos. One of their constructs used the 5′- and 3′-UTR of the *nos* gene to flank the sgRNA; this construct did not yield heritable mutations, though technical aspects such as the low survival rate may in part be responsible for this finding (21).

In summary, our experiments demonstrate that the *S. pyogenes* CRISPR/*cas9* system efficiently cleaves chromosomal DNA in cultured *Drosophila* cells. Our results extend the findings of an independent report (Basset et al. (79)) by a system that features clonally selected cell lines with stable *cas9* expression and that allows for only transient activation of the *cas9* nuclease via transfection of an *in vitro* transcribed guide RNA.

### Integration of PCR-based homologous recombination donors is efficient in *Drosophila* S2 cells

Chromosomal DNA double-strand breaks stimulate DNA repair and this can be used for genome editing via HR. A prominent example of this is the addition of a GFP moiety or other protein tags to the endogenous copy of a given protein-coding gene. To this end, the protein tag sequence and a selectable marker are decorated with flanking arms of homologous sequence, typically in the kilobase range (12,14,15,39,41,80–82), thus specifying the desired site of integration. In analogy to elegant approaches used for protein tagging in budding yeast (64), we tested whether these homology arms can be appended via simple additions to PCR primers. This approach is extraordinarily convenient as no cloning steps are involved; the downside is a potentially much lower efficiency of HR due to the length limitations in oligonucleotide synthesis.

We constructed a series of plasmids that serve as templates during PCR to generate a given HR substrate for C-terminal protein tags. The resistance marker is the blasticidin deaminase (BSD) gene from *Aspergillus terreus* allowing for very rapid selection, driven by the *copia* promoter and flanked by FRT sites to permit removal after selection. The sequence coding for a given epitope tag is inserted directly adjacent to the upstream FRT site using *Xho*I and *Spe*I restriction sites. Note that *Xho*I/*Sal*I and *Spe*I/*Xba*I/*Nhe*I have compatible overhangs that can be used for cloning if one of the original sites is present within the desired tag. If the sequence used for primer annealing during PCR is chosen upstream of the *Xho*I site, a series of HR substrates for introducing different tags can be generated with a single set of homology-containing long oligonucleotides. The general setup and resulting linker sequence are detailed in Figure 3A and the supplementary information. Currently, vectors are available with templates for GFP, 2x FLAG and a Twin-STREP-tag (83).

**Figure 4.**
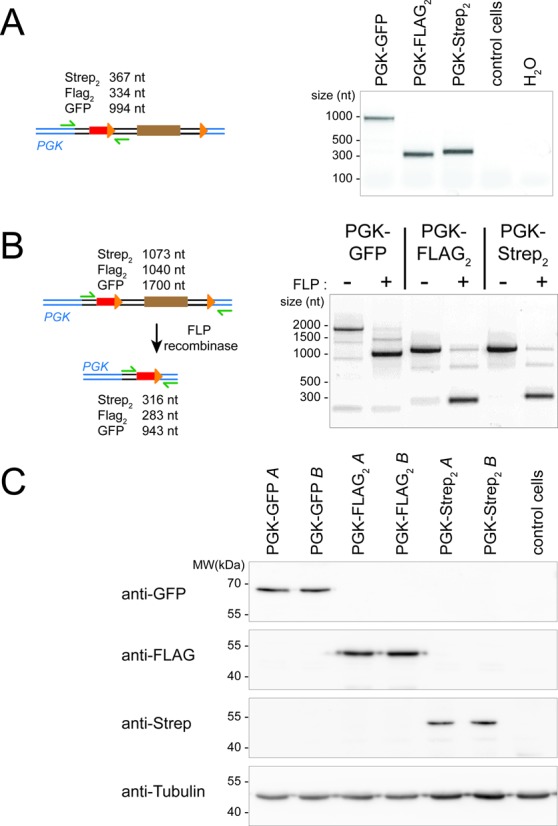
Molecular characterization of epitope tags introduced at the PGK locus. (**A**) PCR analysis to verify integration at the PGK locus. The primer locations and expected product sizes are indicated on the left, the specific upstream and downstream primers were chosen outside of the homology-region used for targeting. The PCR products were sequenced to verify their identity. (**B**) FLP-mediated excision of the resistance cassette. The primer locations and expected product sizes before and after excision are indicated on the left. FLP-mediated excision was further verified by sequencing the PCR products obtained for all three tags after introduction of FLP recombinase. (**C**) Western blot to verify that a defined protein of the expected size is tagged using our protocol. The blots were probed with monoclonal antibodies against GFP (clone B-2 obtained from Santa Cruz), the FLAG-tag (clone M2 obtained from Sigma) and the Strep-tag (Strep-Mab HRP, obtained from IBA in Göttingen/Germany). Detection of tubulin served as a loading control (below, clone E7 obtained from the Developmental Studies Hybridoma Bank). For each tag, two parallel technical replicates were analyzed ( = parallel transfection and selection, referred to as A and B).

We tested the system by tagging the endogenous copy of PGK with GFP. Since the protein is robustly expressed, the fluorescence induced upon successful tagging can be detected and thus conveniently quantified in a flow cytometer. Previous attempts to use HR for genome editing in *Drosophila* have revealed that inactivating the NHEJ pathway via a mutation in the *lig4* gene can greatly increase the proportion of marker insertions at the desired locus. We therefore compared untreated S2 cells stably expressing the *cas9* nuclease to the same cells with prior depletion of *lig4* via RNA interference. The CRISPR RNA directing cleavage 2 nt 5′ to the stop codon in the PGK gene was co-transfected with decreasing amounts of PCR products containing 80 nt and 60 nt of sequence homology upstream and downstream, respectively. We quantified the proportion of GFP positive cells 5 days after transfection, then after one and two weeks of blasticidin selection. Given the limited extent of homologous sequence, we were surprised to see that even in the absence of any selection, up to 2% of the cell population showed detectable GFP expression. This population increased upon blasticidin selection to up to 35%. The amount of co-transfected targeting construct did not appear to be very critical, though a dose of 10 to 30 ng (per well of a 96-well plate) gave the best results. These values were obtained with cells that had been pre-treated with dsRNA to deplete *lig4*. In untreated cells, no more than 6% of the blasticidin-resistant cells were GFP positive after two weeks of selection, demonstrating that NHEJ mediated off-target integration is responsible for at least a portion of the blasticidin-resistant, non-fluorescent cells (Figure 3B). The reproducibility of this success rate is good as similar results were obtained in a parallel experiment with an independent clone of *cas9* expressing cells (Figure 3B) and a fully independent biological replicate (Figure 3C). Correct and full-length integration was verified by genomic PCR (Figure 4A), excision of the blasticidin-resistance cassette upon transfection of a FLP-recombinase expression plasmid (Figure 4B) and western blotting to confirm the predicted size of GFP-, Flag- and Strep-reactive bands (Figure 4C).

**Figure 5.**
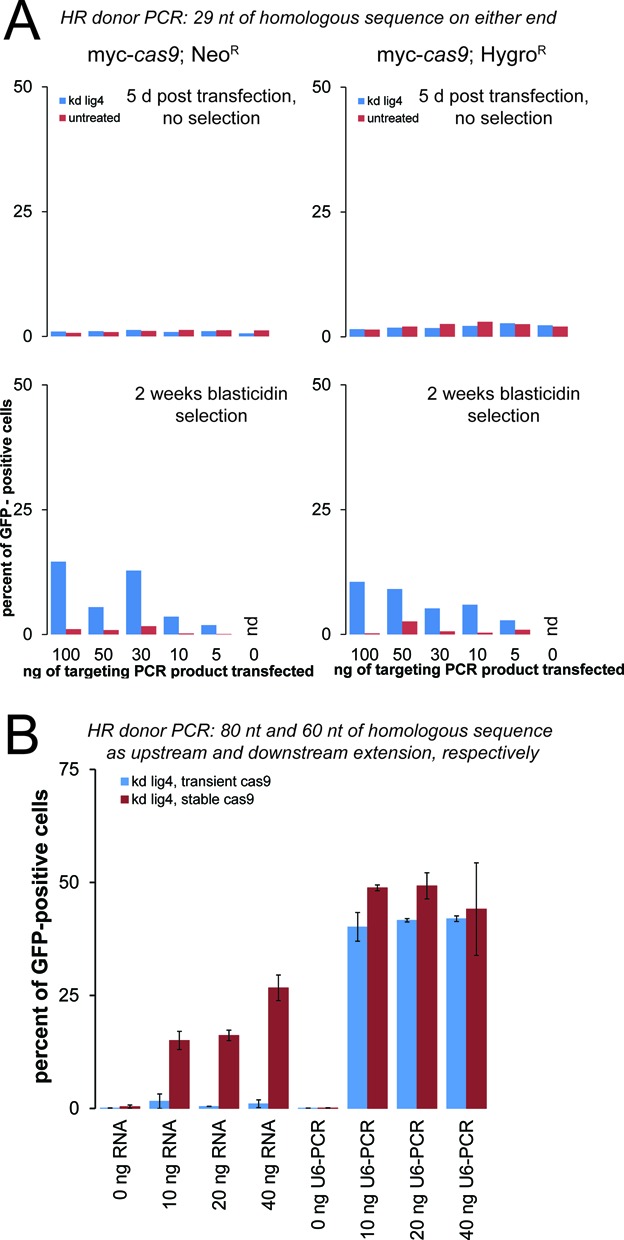
Short homology arms can direct site-specific integration and increased targeting efficiency with U6-promoter based sgRNA expression. (**A**) We performed an analogous experiment to Figure 3B using PCR primers with only 29 nt of sequence homology at either end. Although the efficiency is clearly lower than what was observed with longer homology, the short arms are able to direct site-specific integration of the PCR product. (**B**) Transfection of U6-promoter sgRNA template fusions can further boost the recovery of tag-expressing cells. The amount of sgRNA (obtained by *in vitro* transcription) or U6-promoter-sgRNA template (obtained by overlap extension PCR) is indicated below the diagram. Note that for the *in vitro* transcribed sgRNA, the maximal amount used (40 ng) is less than in Figure 3 (100 ng). Blue bars depict the results from transient *cas9* expression, while red bars depict the results obtained with a stable *cas9* expressing clone (hygromycin-resistance). The bars represent the mean of two fully independent biological replicates, error bars indicate the range of the individual values.

**Figure 6.**
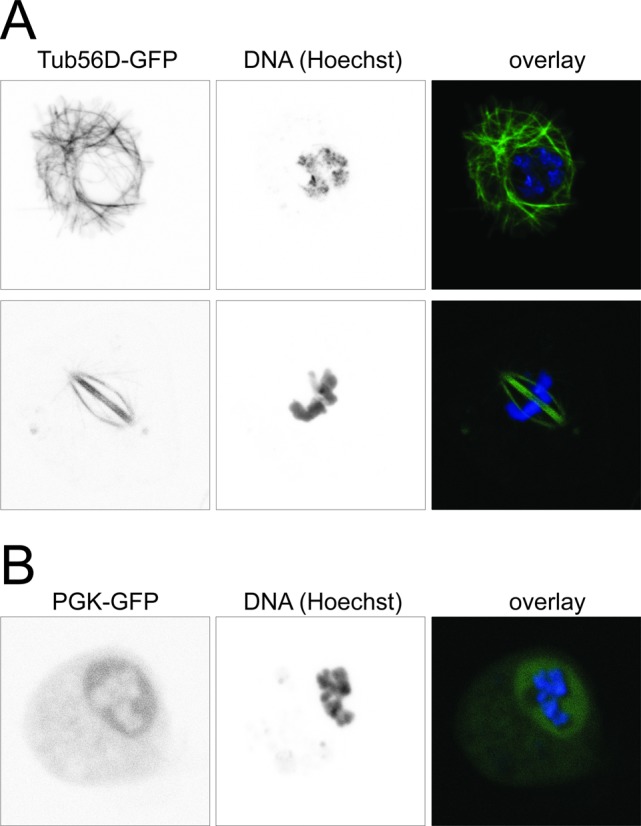
Fluorescence microscopy of Tub56D-GFP and PGK-GFP. (**A**) Tub56D-GFP tagging reveals cytoskeletal structures in interphase cells (top row) and the spindle in mitotic cells (bottom row). (**B**) The GFP-tagged PGK protein is localized to both nucleus and cytoplasm. Live cells were stained with Hoechst 33342, a cell-permeable DNA stain with DAPI-like fluorescence, and imaged on a Leica SP2 confocal microscope. We acquired z-stacks covering the entire thickness of the cells, then calculated the corresponding 2D average projections that are displayed in this figure with the Leica software accompanying the microscope. These high magnification images are representative of the GFP-expressing cells in our cultures. In particular, we did not observe any cells with tubulin-like GFP distribution in the PGK-GFP cultures and *vice versa*.

There are two possibilities why we derive a substantial amount of blasticidin-resistant, GFP-negative cells. Either the PCR cassette spontaneously integrated elsewhere in the genome in spite of our *lig4* depletion, or small mistakes during HR or oligonucleotide synthesis have introduced a shift in the reading frame and thus prevented GFP expression. To distinguish these possibilities, we sequenced the PCR products from Figure 2A directly (i.e. without cloning and selection of individual colonies). The sequencing traces showed only minor signs of sequence heterogeneity, indicating that frameshift mutations are unlikely to account for many of the GFP-negative cells. Off-target integration of the tagging-cassette is therefore a frequent event in the selected population and could—at least in theory—lead to expression of the protein tag on unintended proteins. So far, we have not detected any corresponding signal in our Western blots or in the fluorescence microscopy results (see below, Figure 6). Furthermore, transfection of the GFP tagging cassette in the absence of a corresponding DNA break does not yield significant amounts of GFP-positive cells (see below, Figure 5). The non-expressing cells may have suffered from the mutagenic insertion of the tagging cassette into off-target locations. However, since these cells do not appear to express the tag, this should not compromise conclusions based on detection of the tag.

Is it possible to further shorten the homology arms contributed by the PCR primers? We performed the same experiment with only 29 nt of upstream and downstream homology. Without selection, we only detected background levels of GFP fluorescence, but upon blasticidin treatment up to 15% of the selected cell population became GFP positive. Again, the depletion of *lig4* resulted in a clear increase in GFP-positive cells. The success of this experiment demonstrates that flanking homology as short as 29 nt can suffice to direct site-specific integration at an engineered DNA double-strand break (Figure 5A). We nonetheless recommend using longer stretches of homology as this increases the frequency of targeted integration.

### Improved efficiency using U6-promoter based sgRNA expression

Since 5′-extensions of the guide RNA did not impair cleavage efficiency (Figure 2), we tested whether the T7-promoter sequence contained in our sgRNA *in vitro* transcription templates can be used to append a U6 promoter via overlap extension PCR. To this end, we amplified the *Drosophila* U6-C promoter with the T7 RNA polymerase promoter sequence as an extension after the annotated transcription start site. Upon overlap-extension PCR, an U6-promoter-sgRNA template fusion product of ∼600 nt can be obtained (not shown). We then transfected this construct along with our PGK-GFP targeting PCR product (harboring the longer homology arms) into *lig4*-depleted cells stably expressing the *cas9* nuclease. For a side-by-side comparison, we also included *in vitro* transcribed RNA. In this experiment, we varied the amount of CRISPR guide RNA (or U6-promoter fusion) while keeping the amount of tagging construct constant. Even without *cas9* programming, surviving cells could be recovered after two rounds of blasticidin selection. However, essentially none of these cells showed green fluorescence, further substantiating that the fluorescence we observed is due to on-target integration. Upon activation of DNA cleavage at the PGK locus, we saw that the U6-promoter fusion constructs resulted in even higher proportions of cells with PGK-GFP fusions than transfection of *in vitro* transcribed RNA (Figure 5B). We also combined transient transfection of a *cas9* expression plasmid, the U6-promoter-gRNA template fusion PCR and the PGK-GFP tagging PCR product into *lig4*-depleted, normal S2-cells. This combination also produced PGK-GFP expressing cells albeit with a slightly reduced efficiency. Co-transfection of *in vitro* transcribed gRNA instead of the U6-promoter fusions was clearly less efficient when combined with transient *cas9* expression, presumably because much of the gRNA template is degraded before the *cas9* protein becomes expressed (Figure 5B).

### Application to other genes and introduction of point mutations in HR donors

To confirm the general applicability of our technique, we also designed gRNA and HR donor primers to tag the *β-tubulin 56D* gene. Successful integration of the tagging cassette was confirmed by PCR and western blotting (not shown). Upon introduction of a GFP-tag, cytoskeletal structures could be observed by fluorescence microscopy in interphase cells (Figure 6A, upper row) and the spindle was clearly GFP-labeled in mitotic cells (Figure 6A, bottom row). In contrast, microscopic analysis of PGK-GFP expressing cells revealed a diffuse cytoplasmic and nuclear distribution of GFP fluorescence (Figure 6B). This localization pattern is consistent with results obtained in mammalian cells, where cytoplasmic and nuclear localization of glycolytic enzymes—including PKG—has been observed (reviewed in (84)).

Serial dilution of the blasticidin-resistant cells can be performed to derive cell populations that show uniform expression of the tag. We found this to be straightforward from a starting population where 30% of the cells have the tag integrated at the correct position (see Supplementary Figure S1 for an example clone). This may even yield cells where all chromosomal alleles have been modified (i.e. no untagged protein remains), provided that the protein remains functional or is non-essential.

Stimulation of HR by *cas9*-mediated DNA cleavage depends on the availability of a correspondingly located GG PAM. Ideally, the *cas9*/CRISPR target site is disrupted by integration of the tagging cassette such that neither the HR donor nor the modified genomic locus can be cleaved (see Supplementary Figure S2A for targeting design of *PGK* and *Tub56D* locus). If this ideal design is not possible, cleavage of the donor and modified locus can be prevented by including point mutations (translationally silent if located within the coding sequence) in the flanking homology arms of the PCR primers. On the other hand, this may have adverse effects on the efficiency of HR, in particular because our approach provides only short homology arms. Using the nuclear RNAi-factor *blanks* as target protein, we compared the tagging efficiency for point mutant and wild-type HR donor PCR products for a non-disrupted CRISPR target site. Western Blot Analysis demonstrated that integration of a FLAG-tag was clearly more efficient if the HR donor contained the point mutations (Supplementary Figure S2B). Thus, the *cas9*/CRISPR mediated cleavage may occur a short distance away from the desired integration site, provided that point mutations can be introduced in the HR donor. Further experiments are required to delimit the maximal distance of DNA cleavage from the desired integration site that still results in highly efficient tagging.

## Discussion

### Limitations and further applications of the chromosomal tagging approach

The system we are describing here allows for straightforward generation of tagged proteins at their chromosomal locus. This circumvents well-known problems that are inherent to approaches based on plasmid cloning and transient transfection, such as overexpression at heterogeneous levels and the presence of untagged, endogenous protein. However, some general limitations of protein tags remain. First of all, addition of a tag may interfere with the function of the host protein. With plasmid transfections, this can sometimes be masked by the remaining pool of untagged endogenous protein; functionality of the chromosomally tagged protein should therefore be assessed in cell clones where all chromosomal alleles have been tagged. Furthermore, appending a relatively stable protein such as GFP may influence the host protein's stability, leading to altered expression levels despite an unaltered gene dose.

Some fly genes show heterogeneity at the 3′-end due to splice variants that involve the region coding for the protein C-terminus. Fortunately, these cases are the exception rather than the rule. Here, introduction of a C-terminal tag is not only impractical but in addition could influence the outcome of alternative splicing decisions. It may be possible to introduce tags also internally with our method; however, the current template modules all contain a stop codon at the end of the tag and the FRT sequence, which remains after removal of the resistance cassette, will also introduce stop codons depending on the reading frame.

Off-target cleavage by the *cas9* nuclease must be a concern. We have thus far employed CRISPR target sites that are very close to the stop codon of the host protein. Due to the limited sequence space available for DNA cleavage, the choice of CRISPR sites with minimal off-target cleavage potential is essentially impossible in this situation. Further experiments should test whether the DNA cut may be set at a certain distance to the desired integration site, allowing for at least a minimal choice among potential CRISPR target sites. Finally, the use of truncated guide RNAs is not only efficient (see Figure 2) but has also reduced the off-target cleavage rates in cultured human cells (55).

It is also important to consider when delivery of the guide RNA as a transcript or as a U6-promoter-based DNA template is more appropriate. Recent structural analysis has revealed that RNA-binding is required for conformational activation of the *cas9* nuclease (85,86). This likely explains why expression of apo-*cas9* is non-toxic in cells and transgenic flies; delivery of an *in vitro* transcribed guide RNA therefore only produces a transient pulse of CRISPR/*cas9* cleavage activity. If DNA-based guide RNA templates are employed, the potential for spontaneous integration of this cassette into the genome may lead to permanent cas9 activation and thus a high rate of off-target cleavage events. We suggest using *in vitro* transcribed guide RNAs and clonal selection of tagged cells if these will represent the basis for a larger set of detailed analyses. In contrast, the U6-promoter-guide RNA template fusions are valuable tools for automated high throughput screening approaches.

The combination of *lig4* RNAi, cas9-mediated DNA cleavage, introduction of a PCR-generated HR donor and blasticidin selection can routinely yield cell populations where more than 30% of the cells have a correctly targeted insertion at the desired genomic locus. This is almost within the range of what can be achieved by transient transfections, yet it has the advantage that the endogenous promoter and gene dose are conserved thus avoiding overexpression artifacts. It may be possible to improve the yield of correctly inserted fragments further by optimizing the *lig4* knock-down procedure and/or combining it with knockdown of other NHEJ factors, in particular those responsible for a *lig4*-independent NHEJ-like pathway (87–91).

The tagging at endogenous expression levels evidently limits the scope of our technique to the set of proteins normally present in the S2 cells. Analogous template modules for N-terminal tagging can be devised; they should provide a promoter to force expression of the fusion protein until the selection marker is removed by FLP recombinase. Before marker excision, expression of the targeted genes will thus be constitutive or—depending on the promoter—conditional. Although e.g. interactome studies with a protein that is normally absent from the cellular context are likely of limited value, mis-expression strategies have been successful to identify novel components of e.g. the *Wnt* signalling pathway in flies (92). With a properly engineered readout, genome-scale overexpression may thus represent a valid and feasible screening approach in S2-cells. Our system is also well suited for knock-in of e.g. a luciferase reporter either fused to or in place of the host gene. This permits convenient measurement of gene regulation within the chromosomal context, including the 3′-UTR if FLP-mediated excision of the resistance cassette has been applied. Combining these reporters with genome-wide RNAi screens is then straightforward. In particular, since transient *cas9* expression can suffice to introduce the tags (see Figure 5B), it should be possible to extend the application to other *Drosophila* cell lines, which can also be derived *de novo* from flies with a desired genotype (93).

Are PCR-based HR donors less efficient than the established, plasmid-based approaches using long homologies? We have not attempted a side-by-side evaluation and quantitative comparisons between flies and S2-cells are not reasonable. An earlier attempt to detect genome modification of KC167/M3 cells via HR by the Cherbas lab employed long homology arms, but could not induce a site-specific DNA break (94). It is thus not suitable for direct comparison with our data. The independent work of Basset et al. (79) in S2-cells demonstrated tagging efficiencies for the Ago1 gene of around 1% without selection using a 1 kb homology arm. This is in fact roughly comparable with the efficiencies we observed at the PGK locus prior to selection and without depletion of *lig4* (see Figure 3B). Subsequently, the approach by Bassett et al. only removes the non-transfected cells, increasing the tagging frequency to 4–5% of the selected cells. Our protocol selects for stable integration of a marker and we thus obtained a much higher proportion of cells expressing tagged proteins. Several publications have demonstrated that linear HR donors (66) or circular HR donors with homology arms of less than 200 nt in length are inefficient when micro-injected into fly embryos (63). In contrast, work in cultured mammalian cells demonstrated comparableintegration efficiency for long, cloned and short, PCR-based homology arms (95). It therefore remains to be tested whether our PCR-based HR donors can be used efficiently *in vivo* as well.

In addition to the two supplementary figures mentioned in the text, the manuscript is accompanied by a detailed step-by-step protocol for our procedure that includes a collection of several figures giving technical detail on the constructs presented and information for primer design.

## AVAILABILITY

The plasmids described in this manuscript will be available through Addgene.

## SUPPLEMENTARY DATA

Supplementary Data are available at NAR Online.

SUPPLEMENTARY DATA
