# Supplementary material for: Efficient chromosomal gene modification with CRISPR/cas9 and PCR-based homologous recombination donors in cultured Drosophila cells
Source: Nucleic Acids Res. 2014 Apr 19;42(11):e89. doi: 10.1093/nar/gku289 (PMC4066747; doi:10.1093/nar/gku289)
Supplement: SUPPLEMENTARY DATA [file supp_42_11_e89__index.html]

Efficient chromosomal gene modification with CRISPR/cas9 and PCR-based homologous recombination donors in cultured Drosophila cells — Efficient chromosomal gene modification with CRISPR/cas9 and PCR-based homologous recombination donors in cultured Drosophila cells — SUPPLEMENTARY DATA 

# Efficient chromosomal gene modification with CRISPR/*cas9* and PCR-based homologous recombination donors in cultured *Drosophila* cells

## SUPPLEMENTARY DATA

**Files in this Data Supplement:**

- SUPPLEMENTARY DATA
- SUPPLEMENTARY DATA
