# Supplementary material for: Efficient chromosomal gene modification with CRISPR/cas9 and PCR-based homologous recombination donors in cultured Drosophila cells
Source: Nucleic Acids Res. 2014 Apr 19;42(11):e89. doi: 10.1093/nar/gku289 (PMC4066747; doi:10.1093/nar/gku289)

Supplementary Figures and Legends for Böttcher et al.

Supplementary Figure 1:

FACS analysis of a clonally derived cell line harboring a PGK-GFP tag and, as controls, a PGK-$Flag_2$-tag. Upper part:

Overlay histogram showing the intensity ditraibution for PGK-GFP (green) and PGK-$Flag_2$ (red). The two curves overlap slightly, therefore quantification is performed on two-dimensional dot plots.

Lower part:

Two-dimensional dot-plot for GFP fluorescence intensity (abscissa) and side scatter intensity (ordinate). In this diagram, the two cell populations can be clearly separated.

Supplementary Figure 2:

A) Details on the targeting design for the *PGK* locus (top) and the *Tub56D* locus (bottom). The CRISPR target site (boxed) and a small portion of the homologous sequence included in the primers to generate the HR donor PCR product are shown. PAM: protospacer associated motif; magenta triangles: predicted sites of DNA cleavage by the *cas9* nuclease programmed to target the indicated sequence.

B) Comparison of targeting efficiency in a situation where the HR donor and the modified locus may be cleaved by the cas9 nuclease.

Upper part: Sequence diagram of the PGK locus as in A)

Lower part:

Western Blot analysis of tagging efficiency at the Blanks locus using wild type (left) and point mutant (right) HR donors. Three technical replicates (= parallel transfection and selection) are shown for each condition. Tubulin served as loading control. The tag was detected with the anti-Flag M2 monoclonal antibody (Sigma).

Supplementary Figure 1

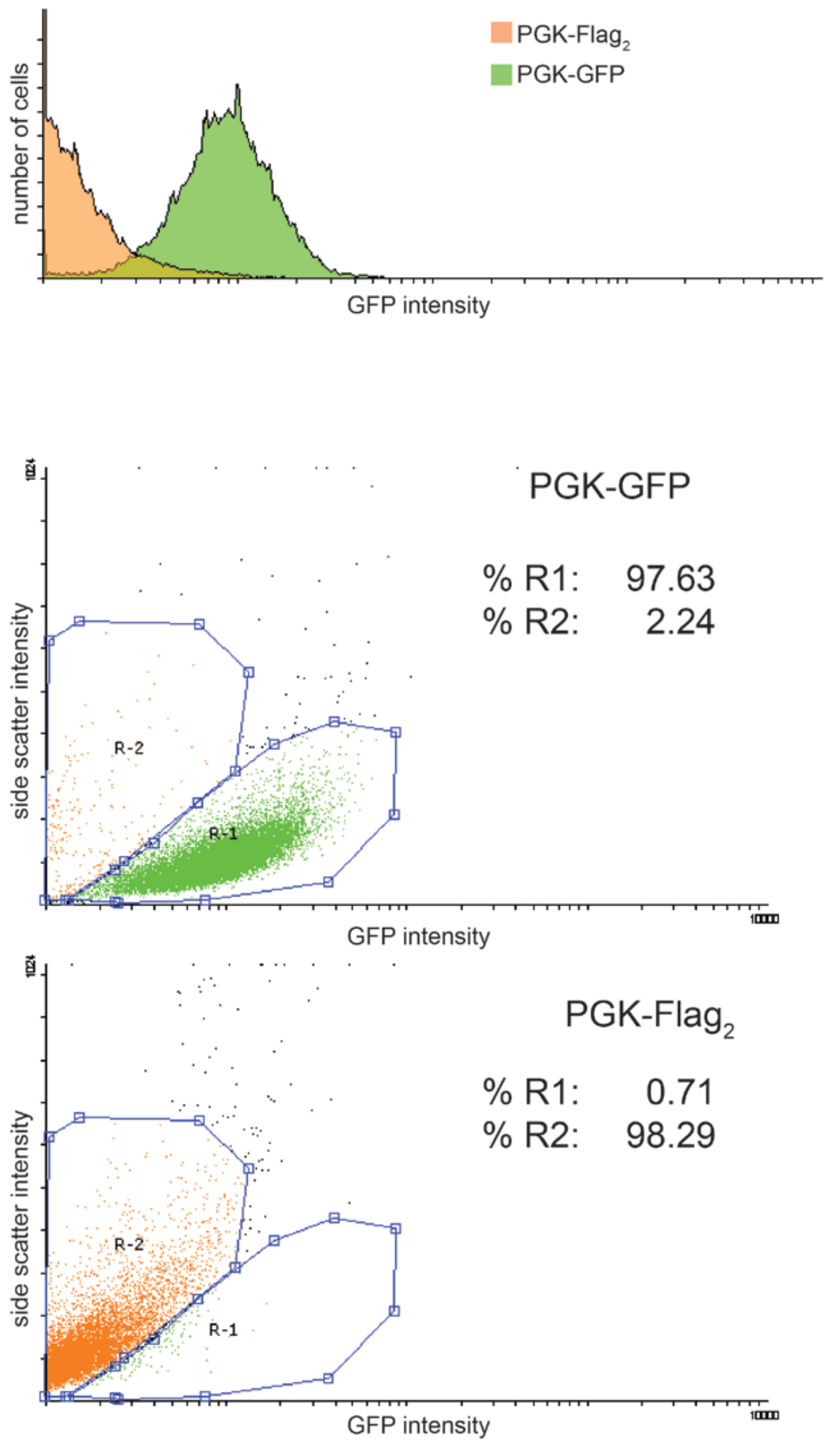

Supplementary Figure 2

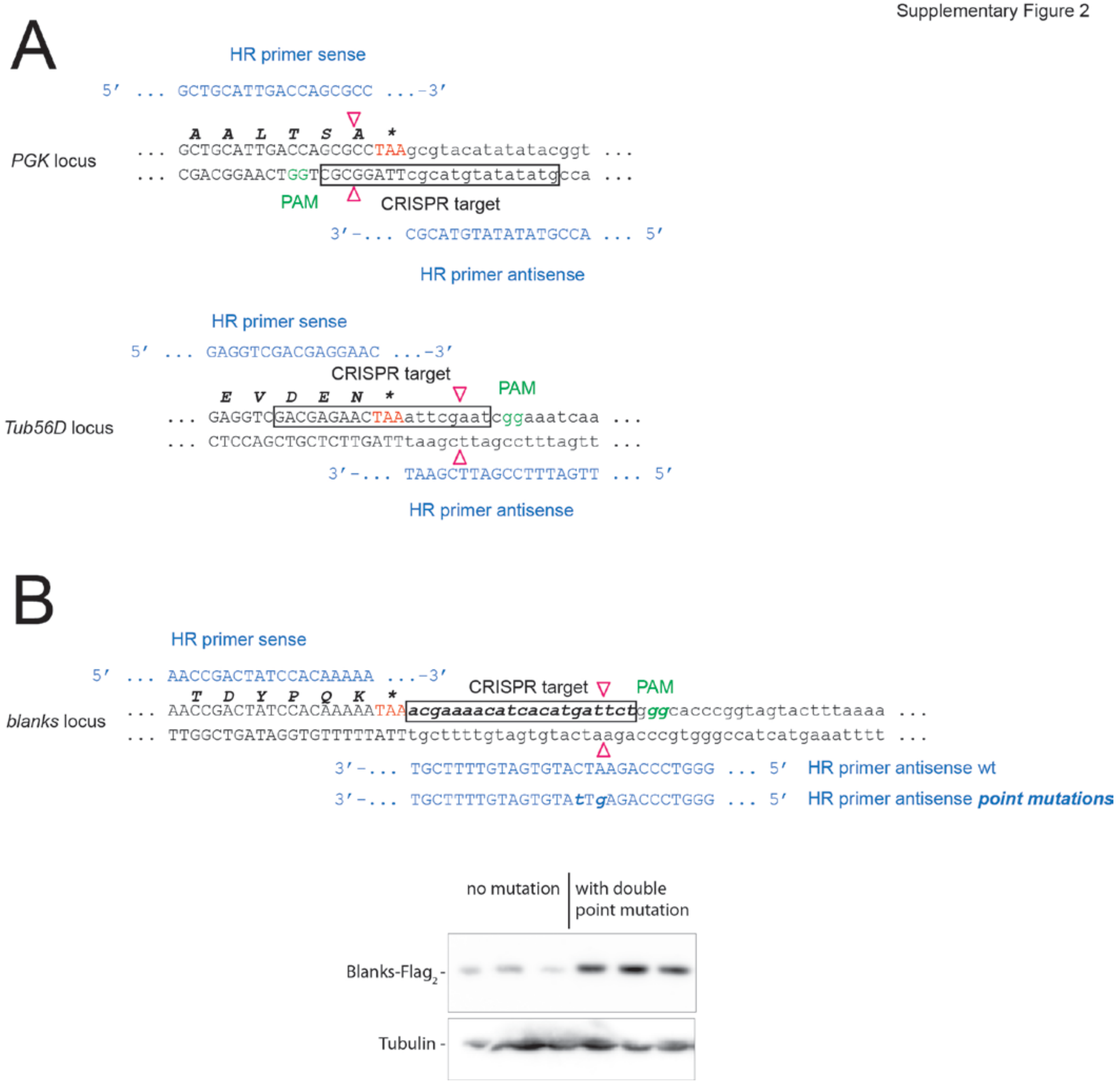

Supplement: SUPPLEMENTARY DATA [file supp_gku289_nar-00510-met-h-2014-File008.pdf]
