# Supplementary material for: Efficient chromosomal gene modification with CRISPR/cas9 and PCR-based homologous recombination donors in cultured Drosophila cells
Source: Nucleic Acids Res. 2014 Apr 19;42(11):e89. doi: 10.1093/nar/gku289 (PMC4066747; doi:10.1093/nar/gku289)
Supplement: SUPPLEMENTARY DATA [file supp_gku289_nar-00510-met-h-2014-File009.pdf]

Genomic Tagging in *Drosophila* cells

This document is a detailed, step-by-step protocol for the introduction of protein tags with PCR-based cassettes. It is broken up into four sections:

Section A: Reagent preparation

Section B: Cell preparation, transfection and selection

Section C: Detection of integration by PCR and Western blot

Section D: Cell cloning

A-1. Generation of a template for sgRNA in vitro transcription

Material:

- general PCR reagents
- Scaffold primer serving as template during PCR (0.1 µM concentration):

5′-GTTTTAGAGCTAGAAATAGCAAGTTAAAATAAGGCTAGTCCGTTATCAACTTGAAAAAGTGGCACCGAGTCGGTGC-3′

- Antisense primer for PCR (10 µM concentration)

5′-GCACCGACTCGGTGCCACT-3′

- Sense primer for PCR containing the specific sequence for programming of the cas9 nuclease (10 µM concentration, specific sequence in upper case letters)

Example: 5′-taatacgactcactataGNNNNNNNNNNNNNNNNNNNNNgttttagagct-3′

A slight modification of the T7 promotor sequence allows generation of sgRNAs with a 5’-A:

Example: 5′-taatacgactcactattANNNNNNNNNNNNNNNNNNNNNgttttagagct-3′

(do *not* use this primer for overlap-extension PCR to append the U6 promotor)

<u>PCR mix for sgRNA template:</u>

5	ul	0.1 uM oligo scaffold
1	ul	10 uM primer targeting
1	ul	10 uM primer antisense scaffold
5	ul	10x PCR buffer (we use Fermentas)
4	ul	25 mM $MgCl_2$
1	ul	10 mM (each) dNTP
32.5	ul	H_2O
0.5	ul	Taq polymerase

<u>PCR program:</u>

1:	94°C	2min.
2:	94°C	20 sec.
3:	50°C	20 sec.
4:	72°C	20 sec.
5:	goto	step 2, 34 repetitions (=35 cycles in total)
6:	4°C	pause

Quality control: Load 5 ul of the PCR on a 2% agarose gel; the PCR product should run at about 110 nt (may vary depending on the length of the targeting region)

Use this PCR directly as template for in vitro transcription without further purification.

A-2. Generation of a the sgRNA for transfection by in vitro transcription

Material:

10x T7 transcription buffer:

500 mM	Tris-HCl pH7.9
25 mM	spermidine
260 mM	$MgCl_2$ (really 260 mM)
0.1%	Triton-X100

100 mM solution of each NTP — These can either be purchased ready-made or prepared from the powder, which is significantly cheaper. You need to adjust the pH to ~7 with NaOH (sticks are fine and help to avoid RNase contamination) after dissolving the salts!

DTT, RNase inhibitor, T7 RNA polymerase, RNase-free DNase-I

IVT recipe:

12.5 ul	PCR product from step 1
5 ul	10 x T7 transcription buffer
2 ul	ATP (100 mM)
2 ul	GTP (100 mM)
2 ul	UTP (100 mM)
2 ul	CTP (100 mM)
0.3 ul	1 M DTT
1 ul	RiboLock (Fermentas)
21.2 ul	H_2O (RNase-free)
2 ul	T7 RNA polymerase

IVT incubation

37°C	>= 3 hours, can be over night;	we use our incubator for bacterial plates for this as it will prevent condensation of water under the lid
add	1ul DNase-I	
37°C	30 min.	
95°C	10 min.	
65°C	20 min.	

Cool to RT, spin to remove the precipitate of Magnesium-pyrophosphate that may have formed.

The RNA should be column-purified for maximum transfection efficiency. We use a Qiagen PCR purification kit for this purpose (treating the IVT as if it were a PCR). The recovery with this approach is only about 50% but that is usually sufficient.

Quality control: Load 1 ul of the purified RNA on a 2% non-denaturing agarose gel. We often observe a doublet band at ~150 and ~300 nt of a DNA size marker; 10% acrylamide-urea gels will give nicer results but are not necessary in our experience. The band should be well visible; we estimate the RNA-amount by comparing the band intensity with the DNA size marker. Spectrophotometric quantification is problematic because even after the purification step a significant amount of free nucleotides remain, leading to overestimation of the RNA quantity.

A-3. Generation of a U6-sgRNA template for transfection by overlap-extension PCR

Material:

- general PCR reagents
- template plasmid pRB17 containing the U6-promotor with a T7 extension
- PCR product for sgRNA in vitro transcription from step 1
- sense primer for PCR (10 µM concentration)

`5'-GCTCACCTGTGATTGCTCCTAC-3'`

- antisense primer for PCR with RNA polymerase III termination sequence (10 µM concentration)

`5'-gcttattctcAAAAAAGCACCGACTCGGTGCCACT-3'`

<u>PCR mix for U6-sgRNA fusion:</u>

1	ul	20 ng/ul pRB17
1	ul	sgRNA template PCR from step 1
1	ul	10 uM primer sense
1	ul	10 uM primer antisense polIII ter.
5	ul	10x PCR buffer (we use Fermentas)
8	ul	25 mM $MgCl_2$
1	ul	10 mM (each) dNTP
32.5	ul	H_2O
0.5	ul	Taq polymerase

<u>PCR program:</u>

1:	94°C	2min.
2:	94°C	20 sec.
3:	50°C	20 sec.
4:	72°C	20 sec.
5:	goto	step 2, 34 repetitions (=35 cycles in total)
6:	4°C	pause

It is important to purify this PCR product prior to transfection because the PCR-buffer usually contains detergents that will negatively affect transfection efficiency. We use the Qiagen PCR purification kit for this purpose.

Quality control: Load 3 ul of the purified product on a 1.2% agarose gel. The band should run at ~600 nt, we sometimes see a smaller band at ~150 nt appearing. If this is the case, try to improve the hot-start conditions.

A-4. Generation of homologous recombination template for tagging by PCR

Material:

- general PCR reagents
- template plasmids for tagging (100 pg/ul dilution), e.g. pMH3 (GFP), pMH4 ($Flag_2$), pIW1 ($Strep_2$)
- sense and antisense primers containing the desired homology regions (10 µM concentration); the length of the homology region can be varied according to the specification of your oligonucleotide synthesis provider. We recommend using at least 80 nt on the sense primer and at least 60 nt on the antisense primer.

e.g. sense (homology region in upper case letters):

```
5'-
CGTCTCCACCGGAGGCGGCGCTTCGCTGGAGCTCCTGGAGGGCAAGACACTGCCAGGCGTGGCTGCATTGACCAG
CGCCggatcttccggatggctcgag-3'
```

e.g. antisense (homology region in uppercase letters):

```
5'-
TGGTTTGTGCTTACAAGGTAAACGATGCGATTAACATTAATATACCGTATATATGTACGCgaagttcctattctc
tagaaagtataggaacttccatatg-3'
```

<u>PCR mix for HR-donor tagging PCR:</u>

1	ul	100 pg/ul template plasmid
1	ul	10 uM primer sense
1	ul	10 uM primer antisense polIII ter.
5	ul	10x PCR buffer (we use Fermentas)
4	ul	25 mM $MgCl_2$
1	ul	10 mM (each) dNTP
36.5	ul	H_2O
0.5	ul	Taq polymerase

<u>PCR program:</u>

1:	94°C	2min.
2:	94°C	20 sec.
3:	50°C	20 sec.
4:	72°C	20 sec.
5:	goto	step 2, 34 repetitions (=35 cycles in total)
6:	4°C	pause

It is important to purify this PCR product prior to transfection because the PCR-buffer usually contains detergents that will negatively affect transfection efficiency. We use the Qiagen PCR purification kit for this purpose.

Quality control: Load 3 ul of the purified product on a 0.8% agarose gel. The final size of the product depends on the particular tag(s) amplified: GFP ~1850 nt, $Flag_2$ ~1150 nt, Strep2 ~1200 nt.

A-5. Generation of dsRNA to deplete *lig4*

Material:
- general PCR reagents
- Primers with T7 extension (sense and antisense, 10 µM concentration)

sense: `5'- taatacgactcactatagggCCCAATGATCCAAAGTGTTTTTGCA-3'`
antisense: `5'- taatacgactcactatagGGAAGTAGGATGCCTTCGCGA-3'`
- Drosophila cDNA as template for PCR
- IVT reagents as described for step 2

PCR mix for dsRNA template:

1	ul	Drosophila cDNA
1	ul	10 uM primer sense
1	ul	10 uM primer antisense
5	ul	10x PCR buffer (we use Fermentas)
4	ul	25 mM $MgCl_2$
1	ul	10 mM (each) dNTP
36.5	ul	H_2O
0.5	ul	Taq polymerase

PCR program:

1:	94°C	2min.
2:	94°C	20 sec.
3:	50°C	20 sec.
4:	72°C	20 sec.
5:	goto	step 2, 34 repetitions (=35 cycles in total)
6:	4°C	pause

Use this product directly as template for in vitro transcription.

IVT recipe:

25 ul	PCR product from above
10 ul	10 x T7 transcription buffer
4 ul	ATP (100 mM)
4 ul	GTP (100 mM)
4 ul	UTP (100 mM)
4 ul	CTP (100 mM)
0.6 ul	1 M DTT
42.4 ul	H_2O (RNase-free)
4 ul	T7 RNA polymerase

IVT incubation

37°C	>= 3 hours, best is over night;	we use our incubator for bacterial plates for this as it will prevent condensation of water under the lid
add	1ul DNase-I	
37°C	30 min.	
95°C	10 min.	
65°C	20 min.	

Cool to RT, spin to remove the precipitate of Magnesium-pyrophosphate that may have formed.

This dsRNA can be added directly to the cell culture medium to induce RNAi by soaking, provided it is sterile.

Quality control: Load 1 ul and 1 ul of a 1:10 dilution on a 1% agarose gel. The band should run at ~600 nt, sometimes we see a certain extent of "laddering" (=multimer sized fragments). Ideally, the 1:10 dilution should still give a well visible band that can be used to estimate the concentration. Spectrophotometric quantification is impossible due to the presence of high amounts of free nucleotides.

B-1. Knock-down of *lig4* and transfection

Material:
- reagents prepared in section A of this protocol
- Fugene HD transfection reagent (e.g. Promega #E2311)
- Schneider's medium (we use Bio&Sell # BS 2.43G02J)
- Fetal bovine serum (FBS) as a cell culture additive; we prepare our medium with 10% FBS.

Day 1: Split a dense culture of S2-cells to a density of *1x10^6 cells/ml* in Schneider's medium with 10% FBS; one well of a 24-well plate (= 500 ul of medium) is usually sufficient at this point. Add dsRNA for induction of *lig4* knock-down to a final concentration of 10 ug/ml.

Day 4: Once the cells are about to reach saturation, split the culture again to *1x10^6 cells/ml* in Schneider's medium with 10% FBS; at this time, an estimation of the amount of cells required for the experiment should be made and the culture expanded accordingly. Repeat the addition of dsRNA corresponding to *lig4* to a final concentration of 10 ug/ml.

Day8: Count the cells and adjust them to a density of *0.5 x 10^6 cells/ml* in Schneider's medium with 10% FBS. Dispense 100 ul of this cell suspension per well of a 96-well plate. The cells are now ready for transfection.

Prepare the transfection mix (amount given per well) according to the type of experiment you are doing. The amounts of Fugene-HD and nucleic acids may need to be adjusted according to your cells and culture conditions. We recommend using the PGK-GFP tagging approach for optimization.

A) Transient expression of cas9, in vitro transcribed sgRNA
10 ul Schneider's medium without FBS
50 ng of in vitro transcribed sgRNA (step A-2)
50 ng of pRB14
50 ng of HR template PCR product (step A-4)
Mix, then add
1 ul Fugene-HD

B) Transient expression of cas9, U6-sgRNA template fusion
10 ul Schneider's medium without FBS
50 ng of U6-sgRNA fusion PCR (step A-3)
50 ng of pRB14
50 ng of HR template PCR product (step A-4)
Mix, then add
1 ul Fugene-HD

Use S2-cells with myc-cas9 integration, clone 9-4 (neomycin-resistant) or 5-3 (hygromycin-resistant) for stable cas9 expression.

C) Stable expression of cas9, in vitro transcribed sgRNA
10 ul Schneider's medium without FBS
100 ng of in vitro transcribed sgRNA (step A-2)
50 ng of HR template PCR product (step A-4)
Mix, then add
1 ul Fugene-HD

D) Stable expression of cas9, U6-sgRNA template fusion
10 ul Schneider's medium without FBS
75 ng of U6-sgRNA fusion PCR (step A-3)
75 ng of HR template PCR product (step A-4)
Mix, then add
1 ul Fugene-HD

Controls: It is a good idea to carry along a positive control (e.g. PGK-GFP or tubulin-GFP) and a negative control for your targeting construct. One suggestion for a negative control is to leave out the sgRNA component. Another negative control should be to leave out the HR donor PCR; this is at the same time a positive control for the Blasticidin-selection later on.

Let the transfection mix stand for 60 minutes, then add the appropriate volume to each cell culture well.

B-2. Blasticidin selection of transfected cells

Material:
- Schneider's medium with 10% FBS
- Blasticidin-S solution 10 mg/ml (e.g. Life Technologies A11139-03)

Day 4 post transfection: Split the cells 1:5 into medium containing 25 ng/ml Blasticidin (=125 ul of 10mg/ml stock for 50 ml of medium);

Note: Most of the cells will die rapidly at this point (1-2 days). If this split is delayed until day 6 after transfection, more cells will survive the selection and the culture recovers better. However, the selected population may show a somewhat lower proportion of cells expressing the tag fused to the intended protein.

Day 11 post transfection: Split the cells again 1:5 into Blasticidin-containing medium. This time, growth in Blasticidin medium should resume almost normally.
Note: In our hands, selection is complete after this split. Since western blot analysis requires some more material, you may find it convenient to directly split the cells up from 96-well to 24-well format plates at this stage. The remainder of the cells after splitting can be used to isolate DNA for PCR analysis; this can give a first indication whether targeted integration has occurred.

C-1. Molecular Analysis: Small-scale isolation of genomic DNA

Material:
- Gel-extraction kit (e.g. Qiagen)
- cultured cells (~50-100 ul)

Procedure:
1. Resuspend cells in culture vessel and transfer 100 ul (50 ul can suffice if the amount is limiting) to an Eppendorf tube
2. Add 300 ul of Qiagen buffer QG to the cells in medium, vortex thoroughly
3. apply the entire sample to a Qiagen spin column from the gel extraction kit and discard the flow-through
4. wash the column with 700 ul of buffer PE and discard the wash
5. centrifuge the empty column 1 min. at full speed to completely dry the matrix
6. transfer column to fresh Eppendorf cup; apply 50 ul of buffer EB to the column, let stand for 1 min., then centrifuge for 1 min. at full speed to recover the eluate

This DNA preparation works well as template in PCR.
Use 2.5 ul of DNA per 25 ul reaction.

C-2. Molecular Analysis: PCR to check for integration

Material:
- purified DNA from selected S2-cells (step C-1)
- general PCR reagents
- primer copia-as: 5′-GTAGGTTGAATAGTATATTCCAACAGCATATG-3′
- gene-specific upstream sense primer (recommended: 200-1000 nt upstream of integration site, sense orientation)

Procedure:
1. Assemble components of the PCR on ice except the Taq polymerase, use 2.5 ul template
2. Start thermocycler and set it to pause with the block at 94°C
3. Add Taq polymerase to PCR and close tube
4. Place tube from ice directly into the hot block of the thermal cycler, run PCR program
5. Analyze 10 ul of the PCR on a 1% agarose gel. The expected band should have this size:

xxxx nt (distance gene specific sense primer – integration site)
+ xxxx nt (size of integrated tag + linker: 740 nt GFP, 82 nt $FLAG_2$, 115 nt $Strep_2$)
+ 79 nt (distance antisense primer – tag)
Total size of PCR product

PCR recipe:

2.5 ul isolated genomic DNA
1 ul 10 uM primer copia as
1 ul 10 uM primer gene specific sense
2.5 ul 10x PCR buffer (we use Fermentas)
2 ul 25 mM $MgCl_2$
0.5 ul 10 mM (each) dNTP
15.5 ul H_2O
0.5 ul Taq polymerase

PCR program:

1: 94°C 2min.
2: 94°C 20 sec.
3: 55°C 20 sec.
4: 72°C 60 sec.
5: goto step 2, 34 repetitions (=35 cycles in total)
6: 4°C pause

Possible modifications:

- use a hot-start enzyme if you do not wish to preheat the thermocycler
- for longer tags (GFP) and a relatively long distance to the upstream primer, 90 sec. extension time at 72°C may give better results

C-3. Molecular Analysis: PCR to check for FLP-out of marker

Material:

- purified DNA from selected S2-cells (step C-1)
- general PCR reagents
- primer tags_common sense: `5'-ggatcttccggatggctcgag-3'`
- gene-specific downstream antisense primer (recommended: 200-1000 nt downstream of integration site, antisense orientation)

Procedure:

1. Assemble components of the PCR on ice except the Taq polymerase, use 2.5 ul template
2. Start thermocycler and set it to pause with the block at 94°C
3. Add Taq polymerase to PCR and close tube
4. Place tube from ice directly into the hot block of the thermal cycler, run PCR program
5. Analyze 10 ul of the PCR on a 1% agarose gel. The expected band should have this size:

  xxxx nt (distance gene specific antisense primer – integration site)
+ xxxx nt (integrated tag/linker/single FRT: 781 nt GFP, 121 nt $FLAG_2$, 155 nt $Strep_2$)
____
Total size of PCR product

PCR recipe:

2.5 ul	isolated genomic DNA
1 ul	10 uM primer tags_common sense
1 ul	10 uM primer gene specific antisense
2.5 ul	10x PCR buffer (we use Fermentas)
2 ul	25 mM $MgCl_2$
0.5 ul	10 mM (each) dNTP
15.5 ul	H_2O
0.5 ul	Taq polymerase

PCR program:

1:	94°C	2min.
2:	94°C	20 sec.
3:	55°C	20 sec.
4:	72°C	60 sec.
5:	goto	step 2, 34 repetitions (=35 cycles in total)
6:	4°C	pause

Possible modifications:

- use a hot-start enzyme if you do not wish to preheat the thermocycler
- for longer tags (GFP) and a relatively long distance to the upstream primer, 90 sec. extension time at 72°C may give better results

- the combination of gene-specific sense and antisense primers (for C-2 and C-3) can also be used to check if all chromosomal alleles have been tagged. Since the unmodified locus will always produce a small PCR product, and is thus in a competitive advantage during PCR, any cell clone that produces only a product corresponding to the modified locus is very likely a clone with modification of all available alleles.

C-4. Molecular Analysis:

Western Blot to check for tag expression

Numerous Western blot protocols are available and whatever system is up-and-running in your lab will likely do the job. Therefore, this section is more a collection of items that you may find useful as additions or alternatives to established procedures.

1) Sample preparations: For a quick check it is not necessary to make high-quality protein extracts; we harvest 50-100 ul of cells by centrifugation, aspirate the supernatant as much as possible to remove the serum-containing medium (consider using a suction device) and then resuspend & boil the cell pellet in 50 ul of 1x SDS sample buffer. Prior to loading, the samples are centrifuged for 5 minutes at top speed to pellet any non-solubilized material. This procedure generates quite reproducible and sufficiently concentrated extracts. Target proteins expressed at very low expression levels may nontheless require some sort of specific extraction and concentration protocols prior to Western blot detection.

2) Transfer type: We routinely use a wet-transfer with Towbin buffer containing 10% Ethanol (instead of the 20% methanol) in a Bio-Rad chamber with electric tension set to 100 V. This gives good transfer results in 60 minutes, for proteins >100 kDa we increase transfer time to 75 or 90 minutes (this may require some optimization for each target protein).

3) Incubation with primary antibody: We dilute the primary antibodies (Flag: M2 / Sigma, GFP: B-2 / Santa Cruz, Strep: Strep-Mab HRP / IBA) 1:5000 in TBS with Tween-20 and 5% milk. For the anti-Flag M2, blocking/incubating/washing with 0.02% Tween gives excellent results, while for the GFP and Strep-Mab antibodies 0.1% Tween is preferable to avoid higher background staining. We find that incubation with the primary antibody over night in a cold room (e.g. in a 50 ml conical tube on a roller) **significantly** increases signal strength compared with 120 minutes incubation at room temperature. The anti-Flag M2 monoclonal antibody also weakly recognizes one ~130 kDa and ~70 kDa endogenous Drosophila protein. Therefore, a control lane with untreated S2-cells is essential (and for the other tags a good idea, too).

4) Just a reminder if you are using the cell lines with stable cas9 expression: This construct has a myc-tag, therefore we do not recommend the use of myc-tag cassettes for other proteins in conjunction with our cell lines or vector for cas9-expression.

D-1. Cloning of cells expressing tagged proteins

Cell cloning can be done either with the Blasticidin-resistant population (add the antibiotic to the culture medium in this case) or after the marker cassette has been FLP'ed out upon transient transfection of the pMH5 vector (in this case the medium must not contain Blasticidin).

1. Add 100uL of selection media per well to a 96 well plate, leaving the first three colmuns empty. Including 20% of conditioned medium (= "old", sterile-filtered cell culture medium) usually improves the yield.
2. Count the cells that you are cloning and adjust the density to 8000 (=8x10^3) cells / ml. Dispense 125 ul of this into each well of the first three columns of the 96 well plate. This corresponds to plating 1000 cells per well.
3. Using an eight channel multipipetter, transfer 25 uL from column 1 to column 4, from 2 to 5 and from 3 to 6. Then repeat this scheme for the rest of the plate. In the end, you have made three successive steps of 5-fold dilutions, with three columns (24 wells) per dilution step.
4. Wrap a strip of parafilm around the plate and incubate at 25°C for at least two weeks. (*Note:* Continue culturing the pool of selected cells in parallel in case no colonies develop.)
5. Check wells to see if colonies are developing. You may have to wait up to three weeks to easily see colonies by eye. If none have developed after three weeks, repeat selection with the pool of selected cells, starting with 5000 cells per well.
6. Note the position of isolated cell clumps with a waterproof pen on the bottom of the dish. This helps to find it again under the cell culture hood and to remove each colony in about 10 uL of medium with a P20 pipette. First, transfer it to a fresh well of a 96-well plate containing 100 ul of medium.
7. When clonal cell in the 96 well plate are getting dense, dilute them into 24 well plates. When these wells reach high density, split cells again and incubate the new plate at 25°C as before. The old plate can then be used for molecular analysis as described in section C.
8. Amplify the clones of interest further in 6-well plates and freeze aliquots. To be really sure that the population is clonal (in cases where this is essential), we suggest performing two rounds of dilution & selection.

Oligo-Design for Targeting PCR

C-terminal tag or truncated protein with tag:

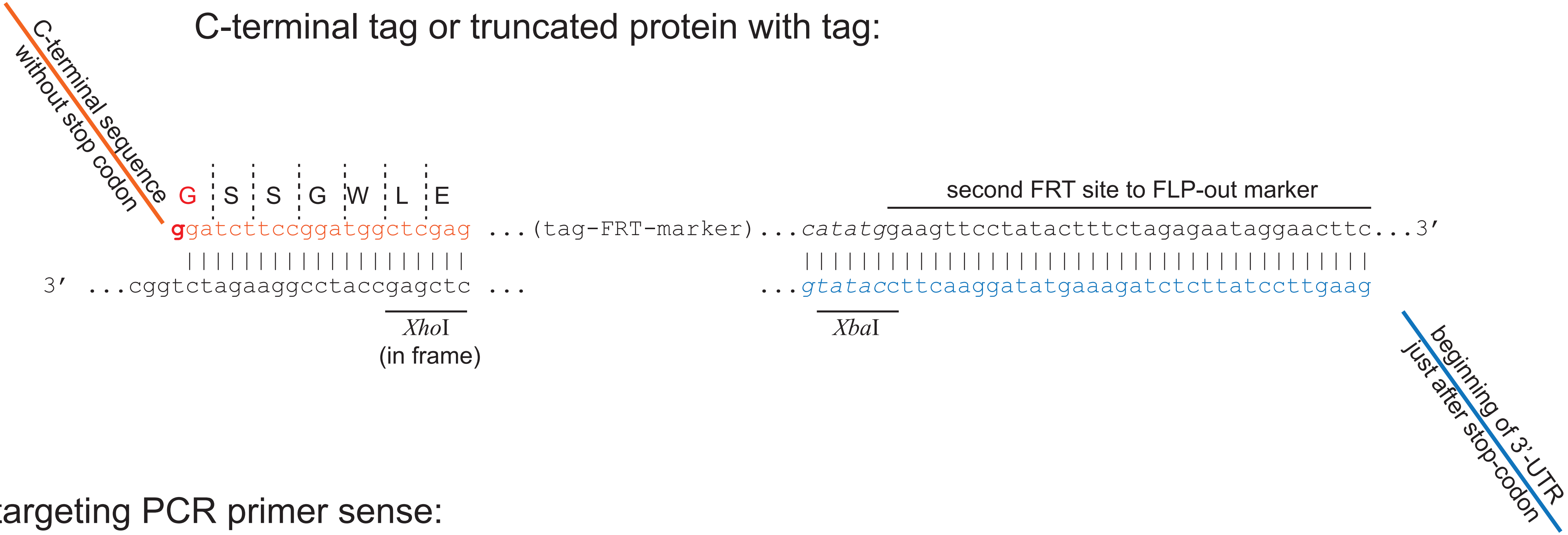

targeting PCR primer sense:

5’-nnnnnnnnnnnnnnnnnnnnnnnnnnnnnnnnnnnnnnnnnnnnnnnnnnnnnnnnnnnnnnnnnnnnnnnnnnnnnnnn**g**gatcttccggatggctcgag-3’

C-terminal sequence of gene without stop codon, ***sense***
(we recommend using >= 80 nt here)

targeting PCR primer antisense:

5’-nnnnnnnnnnnnnnnnnnnnnnnnnnnnnnnnnnnnnnnnnnnnnnnnnnnnnnnnnnnngaagttcctattctctagaaagtataggaacttccatatg-3’

beginning of 3’-UTR downstream of stop codon, ***antisense***
(we recommend using >= 60 nt here)

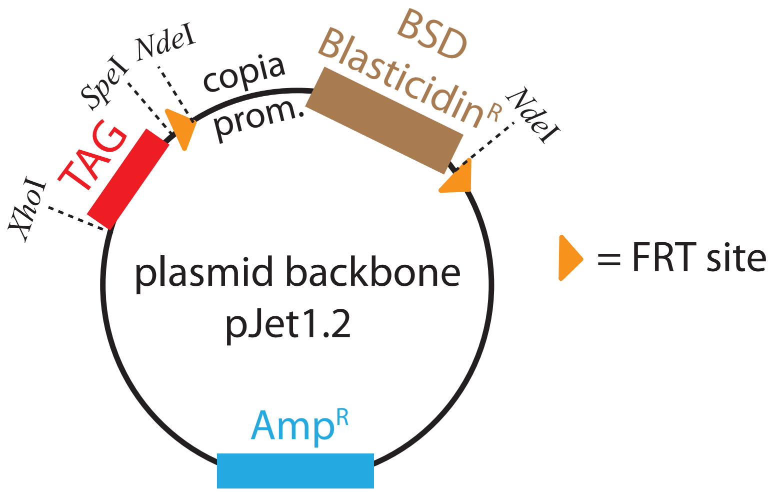

pMH4 based:

```
    G  S  S  G  W  L  E  D  Y  K  D  D  D  D  K  S  G  A  D  Y  K  D  D  D  D  K  *
...ggatcttccggatggctcgagGATTATAAAGATGATGATGATAAAtccggagccGATTATAAAGATGATGATGATAAATGAactagtGAAGTTCCTATACTTTCTAGAGAATAGGAACTTCaaataaacatatgCTGTTG...
                   XhoI                     2x FLAG-tag                       SpeI            FRT site                      NdeI
```

pIW1 based:

```
    G  S  S  G  W  L  E  S  A  W  S  H  P  Q  F  E  K  G  G  G  S  G  G  G  S  G  G  G  S  W  S  H  P  Q  F  E  K  *
...ggatcttccggatggctcgagAGCGCTTGGAGCCACCCGCAGTTCGAGAAAggtggaggttccggaggtggatcgggaggtggatcgTGGAGCCACCCGCAGTTCGAAAAATGAactagtGAAGTTCCTATACTTTCTAGAGAATAGGAACTTCaaataaacatatg...
                   XhoI                                       twin STREP-tag                                  SpeI            FRT site                      NdeI
```

pMH3 based:

```
    G  S  S  G  W  L  E  M  V ...          (EGFP)            ...  Y  K  *
...ggatcttccggatggctcgagATGGAG...       (720 nt EGFP CDS)     ...TACAAGTAAactagtGAAGTTCCTATACTTTCTAGAGAATAGGAACTTCaaataaacatatgCTGTTG...
                   XhoI                                                  SpeI            FRT site                      NdeI
```

CRISPR RNA ivt template oligo design and PCR

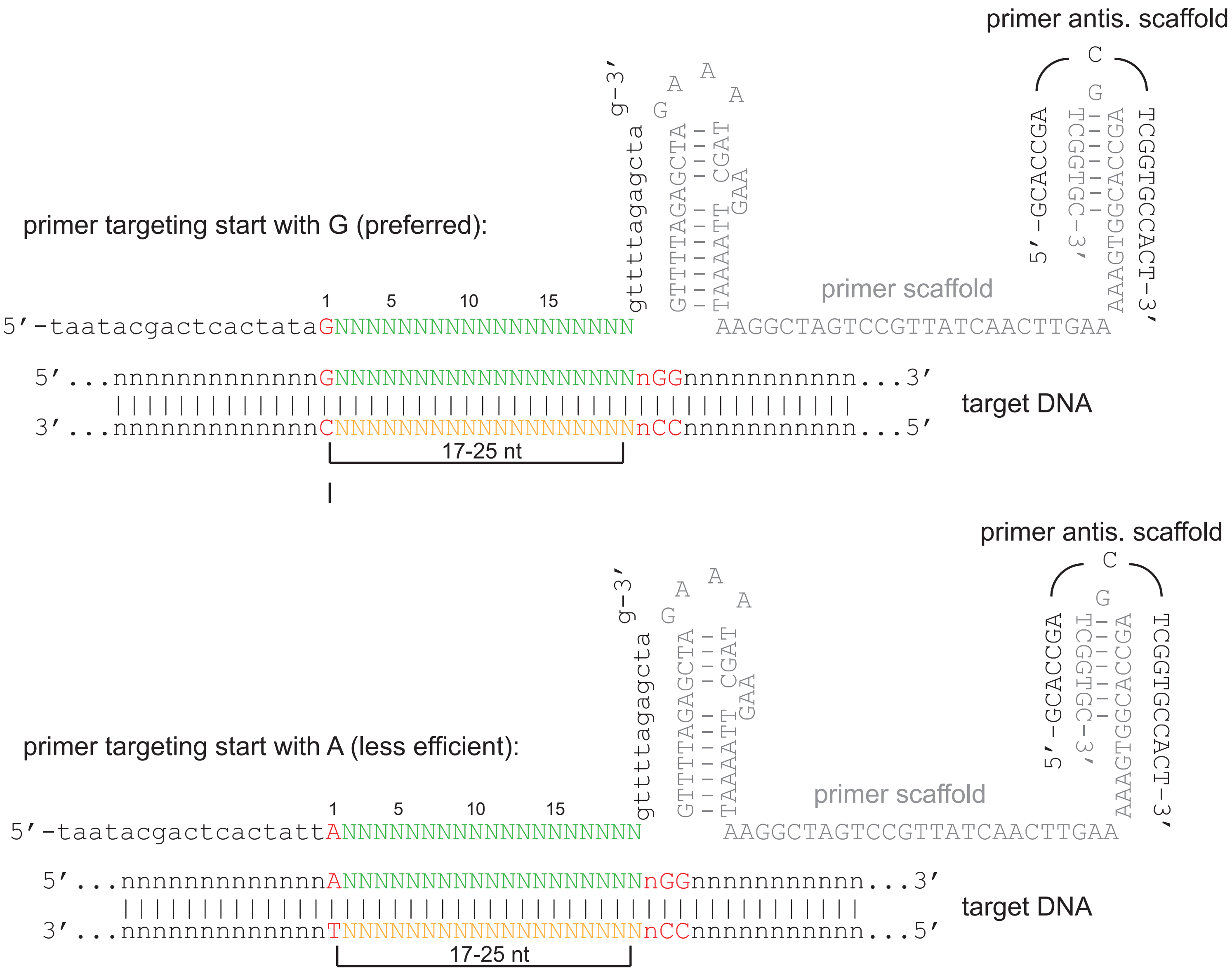

primer targeting start with G:

5′-taatacgactcactataGNNNNNNNNNNNNNNNNNNNNgttttagagctag-3′

primer ivt targeting start with A:

5′-taatacgactcactattANNNNNNNNNNNNNNNNNNNNgttttagagctag-3′

primer scaffold:

5′-GTTTTAGAGCTAGAAATAGCAAGTTAAAATAAGGCTAGTCCGTTATCAACTTGAAAAAGTGGCACCGAGTCGGTGC-3′

primer antis. scaffold:

5′-GCACCGACTCGGTGCCACT-3′

Checklist for Primer Design

CRISPR targeting RNA:

- ○ CRISPR site within region used for homology targeting?
  (if yes, introduce point mutation into homology arm to prevent cleavage)
- ○ PAM correctly identified (GG or CC, if necessary AG or CT)?
- ○ Correct distance (1nt) between CRISPR targeting end and PAM?
- ○ Correct overall length of CRISPR RNA targeting part (17-25 nt)?

Targeting PCR:

- ○ Stop codon excluded?
- ○ If CRISPR site is contained within one homology region:
  Point mutation introduced?
- ○ uspream homology in sense,
  downstream homology in antisense orientation?
